# Santa Ana Winds of Southern California Impact PM_2.5_ With and Without Smoke From Wildfires

**DOI:** 10.1029/2019GH000225

**Published:** 2020-01-11

**Authors:** Rosana Aguilera, Alexander Gershunov, Sindana D. Ilango, Janin Guzman‐Morales, Tarik Benmarhnia

**Affiliations:** ^1^ Scripps Institution of Oceanography University of California San Diego La Jolla CA USA; ^2^ Department of Family Medicine and Public Health University of California San Diego La Jolla CA USA; ^3^ School of Public Health San Diego State University San Diego CA USA

**Keywords:** PM_2.5_, air quality, Santa Ana winds, wildfire smoke, Southern California

## Abstract

Fine particulate matter (PM_2.5_) raises human health concerns since it can deeply penetrate the respiratory system and enter the bloodstream, thus potentially impacting vital organs. Strong winds transport and disperse PM_2.5_, which can travel over long distances. Smoke from wildfires is a major episodic and seasonal hazard in Southern California (SoCal), where the onset of Santa Ana winds (SAWs) in early fall before the first rains of winter is associated with the region's most damaging wildfires. However, SAWs also tend to improve visibility as they sweep haze particles from highly polluted areas far out to sea. Previous studies characterizing PM_2.5_ in the region are limited in time span and spatial extent, and have either addressed only a single event in time or short time series at a limited set of sites. Here we study the space‐time relationship between daily levels of PM_2.5_ in SoCal and SAWs spanning 1999–2012 and also further identify the impact of wildfire smoke on this relationship. We used a rolling correlation approach to characterize the spatial‐temporal variability of daily SAW and PM_2.5_. SAWs tend to lower PM_2.5_ levels, particularly along the coast and in urban areas, in the absence of wildfires upwind. On the other hand, SAWs markedly increase PM_2.5_ in zip codes downwind of wildfires. These empirical relationships can be used to identify windows of vulnerability for public health and orient preventive measures.

## Key Points

- Santa Ana winds have a predominant ventilation effect as background PM_2.5_ is transported offshore from highly polluted areas
- A polluting effect occurs when SAWs spread smoke PM_2.5_ from wildfires inland toward the coastal region
- Statistical approaches that relate surface wind and PM_2.5_ over space and time can help in identifying wildfire PM_2.5_

## 1 Introduction

Fine particulate matter with aerodynamic diameter <2.5 μm (PM_2.5_) can be inhaled into the deepest recesses of the lungs and cause both short‐ and long‐term effects on human health, particularly for respiratory and cardiovascular diseases (Franklin et al., 2015; Liu et al., 2015; Pope & Dockery, 2006). Sources of primary ambient PM_2.5_ include fuel combustion of motor vehicles and industrial facilities, as well as power generation and residential heating. In addition, secondary PM_2.5_ aerosols can be formed in the atmosphere from gases such as sulfur and nitrogen oxides and volatile organic compounds (Chen et al., 2010; Wilson & Suh, 1997). Secondary aerosols can account for more than 50% of the total PM_2.5_ mass, though it varies greatly among regions and seasons (Finn et al., 2008). PM_2.5_ from anthropogenic sources in the United States, including Southern California (SoCal), has decreased in the past decades due to policy implementation (Lurmann et al., 2015; McClure & Jaffe, 2018).

PM_2.5_ also results from biomass burning and is the main component of wildfire smoke with the biggest impact on public health related to short‐term exposure (Gan et al., 2017; Gupta et al., 2018; Liu et al., 2015; McClure & Jaffe, 2018). In the United States, McClure and Jaffe (2018) observed a downward trend in PM_2.5_ during the last three decades, except in regions that were prone to wildfires. Furthermore, recent studies have predicted that wildfire‐specific PM_2.5_ and the associated health burden will increase with a changing climate (Ford et al., 2018; Liu et al., 2016), contrasting with reduced PM_2.5_ emissions from other sources.

Strong winds have a major role in the transport and dispersion of PM_2.5_, since fine particles can remain airborne for weeks and travel distances of hundreds to thousands of kilometers (WHO, 2006; Tai et al., 2010; Wilson & Suh, 1997). In SoCal, Santa Ana winds (SAWs) are episodic pulses of northeasterly, downslope, offshore flow associated with very dry air accelerating and adiabatically warming and drying over the lee (southwestward sloping) slopes of the coastal topography (Guzman‐Morales et al., 2016; Hughes & Hall, 2010; Raphael, 2003). The onset of SAW, after the dry and long warm season and before the first rains of winter, is associated with the traditional wildfire season in the coastal foothills (Westerling et al., 2004) and downwind pulses of PM_2.5_ impacting populated areas on the coast (Phuleria et al., 2005; Wu et al., 2006; Kochi et al., 2016). Anecdotally, SAWs, which typically occur without a surface thermal inversion, under clear skies and without wildfire, have also been related to the best seasonal visibility as they sweep pollution offshore and far out to sea (Corbett, 1996). Quantifying how SAW influence the spatial and temporal variability of PM_2.5_, with and without wildfire smoke, can provide insight on which areas are the most impacted in terms of exposure to air pollution.

Studies characterizing spatial and temporal patterns of PM_2.5_ in SoCal (Choi et al., 2013; Kim et al., 2000a, 2000b) have observed that PM_2.5_ is abundant under fall stagnation conditions, whereas coarse particles of crustal components typically prevail during high SAW conditions in late fall and winter (Guazzotti, 2001; Qin et al., 2012). In addition, SAW events in the fall have been related to biomass burning PM_2.5_ (Qin et al., 2012), emanating from the coastward sloping wildland where vegetation is most abundant and the SAWs are the strongest, and causing substantive health impacts downwind (Delfino et al., 2009). The existing body of work characterizing PM_2.5_ and referencing SAW is limited in time span and spatial extent and has either addressed only a single event or short time series at a limited set of sites. To our knowledge, no study comprehensively and explicitly assessed the variability of PM_2.5_ as influenced by SAW in the absence and presence of wildfires burning upwind.

We expect SAW to benefit air quality along the coast in the absence of wildfires upwind and to have a detrimental effect when wildfires occur. Furthermore, this potential dichotomous relationship between SAW and PM_2.5_ could help identifying the timing and extent of exposure to wildfire PM_2.5_. Here we study the space‐time relationship between daily levels of PM_2.5_ in Southern California and Santa Ana winds spanning 1999–2012 and aim to identify the impact of wildfire on this relationship. This work resolves hundreds of SAW and dozens of wildfire events at a fine spatial resolution over SoCal for an unprecedented time span of 14 years. We adopt a rolling correlation approach as a tool for the identification of wildfire‐related spikes of PM_2.5_ by means of the observed relationship with surface wind. We include a case study of the October 2007 firestorm, composed of over two dozen wildfires in SoCal, to further illustrate the effect of SAW on PM_2.5_.

## 2 Data and Methods

### 2.1 SAW and SAWRI

The SAW season extends from October to April, peaking in frequency in December and January, when it is not uncommon for up to half of the days to experience Santa Ana conditions (Guzman‐Morales et al., 2016). SAW episodes can also occur every other September and May, on average (Guzman‐Morales et al., 2016), and we therefore also consider these months in our analysis.

The original gridded hourly near‐surface winds from the California Regional dynamical downscaling of a global reanalysis product to 10 × 10 km (CaRD10; Kanamitsu & Kanamaru, 2007) were previously analyzed to derive SAW indices and validate them against the available in situ hourly wind observations by Guzman‐Morales et al. (2016). The daily version of the original hourly SAW regional index (SAWRI), which represents the mean wind speed (m/s) evaluated over the Santa Ana wind domain (Guzman‐Morales et al., 2016), is used here (Figure S1 in the supporting information). SAWRI thus provides an observationally validated regional daily summary of the dynamically downscaled SAWs.

### 2.2 PM_2.5_ Data and Exposure Estimates

The study area comprised 578 zip code polygons whose centroids fell within the SAW domain (Figure S2 in the supporting information). Daily‐, zip code‐specific concentrations of PM_2.5_ were estimated from 1999 to 2012 using 24‐hr daily means sampled and analyzed by the US EPA Air Quality System (https://www.epa.gov/aqs). We used daily PM_2.5_ measured with the Federal Reference Method from ground monitoring stations within a 20 km radius of each population‐weighted zip code centroid. Values were interpolated using an inverse distance weighting approach, where the measured concentration is weighted by the inverse distance squared to each point of interest; this gives greater importance to values reported by monitoring stations closer to the point of interest than monitoring stations farther away in distance.

Interpolated values at each population‐weighted centroid were then assigned to each zip code for daily‐, zip code‐specific concentrations of PM_2.5_. To validate interpolated estimates, each data point was removed and then predicted at that location using remaining data points. Correlations between predicted and actual values at the location of the omitted point were used to assess validity of modeled estimates (*r* = 0.55; Figure S3). ArcMap10.3 (ESRI, 2015) was used to assign buffers, interpolate, and validate. Data management was conducted in SAS 9.3 (SAS, 2012).

### 2.3 PM_2.5_ Anomalies Weighted by SAWRI: Rolling Correlation Between Air Quality and SAW

We calculated daily anomalies (i.e., deviation from the mean) for PM_2.5_ by obtaining the mean within a centered 31‐day window and for each of the 578 zip codes previously selected for being located within the SAW domain, and then subtracting this evolving mean from the original daily PM_2.5_ value. Lastly, we weighted the anomalies according to the strength of SAW, by multiplying by SAWRI and thus reflecting the contribution of strong SAWs in the absence and presence of wildfire events. We also thus obtained values of PM_2.5_ weighted anomalies for the days when SAWs were active in the region. We correlated the weighted anomalies of PM_2.5_ with SAWRI for centered rolling windows of 31 days, moving forward at 1 day increments, for each zip code. The rolling window approach allowed us to visualize and quantify the change in correlation over time and with an emphasis on PM_2.5_ during strong SAWs, which can signal events such as wildfire smoke. Significant positive and negative Pearson correlations were identified, based on the *t* test statistic with 95% confidence. We also tested 15‐ and 60‐day windows and observed similar predominant patterns in terms of positive and negative correlations. Results presented in the following sections correspond to the 31‐day sliding window. All the above analyses were conducted in R version 3.5.1. (R Core Team, 2018).

### 2.4 Case Study Setting: October 2007 Firestorm

More than two‐dozen fires broke out between 20 and 23 October, driven by strong SAWs and burning over 972,000 acres across SoCal. Adding to the size and extent of these fires was the historic 2006–2007 drought that contributed to high dead fuel loads (Keeley et al., 2009). Approximately 3,200 structures were destroyed and several were damaged, causing 161 injuries (particularly among firefighters) and 7 fatalities (Keeley et al., 2009). The property damages amounted to $1.8 billion (Karter, 2008) and were concentrated in San Diego County (83%) followed by San Bernardino County (14%; Keeley et al., 2009).

In San Diego County, Hutchinson et al. (2018) found that respiratory diagnoses, especially asthma, were elevated for vulnerable populations such as children and adults with low income. In the same region, Thelen et al. (2013) observed that at peak fire PM (both coarse and fine) concentrations the odds of a person seeking emergency care increased by ~50% with respect to nonfire conditions. Overall, the 2007 SoCal wildfires imposed an estimate of $3.4 million in healthcare costs for hospital and emergency department visits alone (Kochi et al., 2016).

## 3 Results

### 3.1 Descriptive Statistics

**Figure 1:**
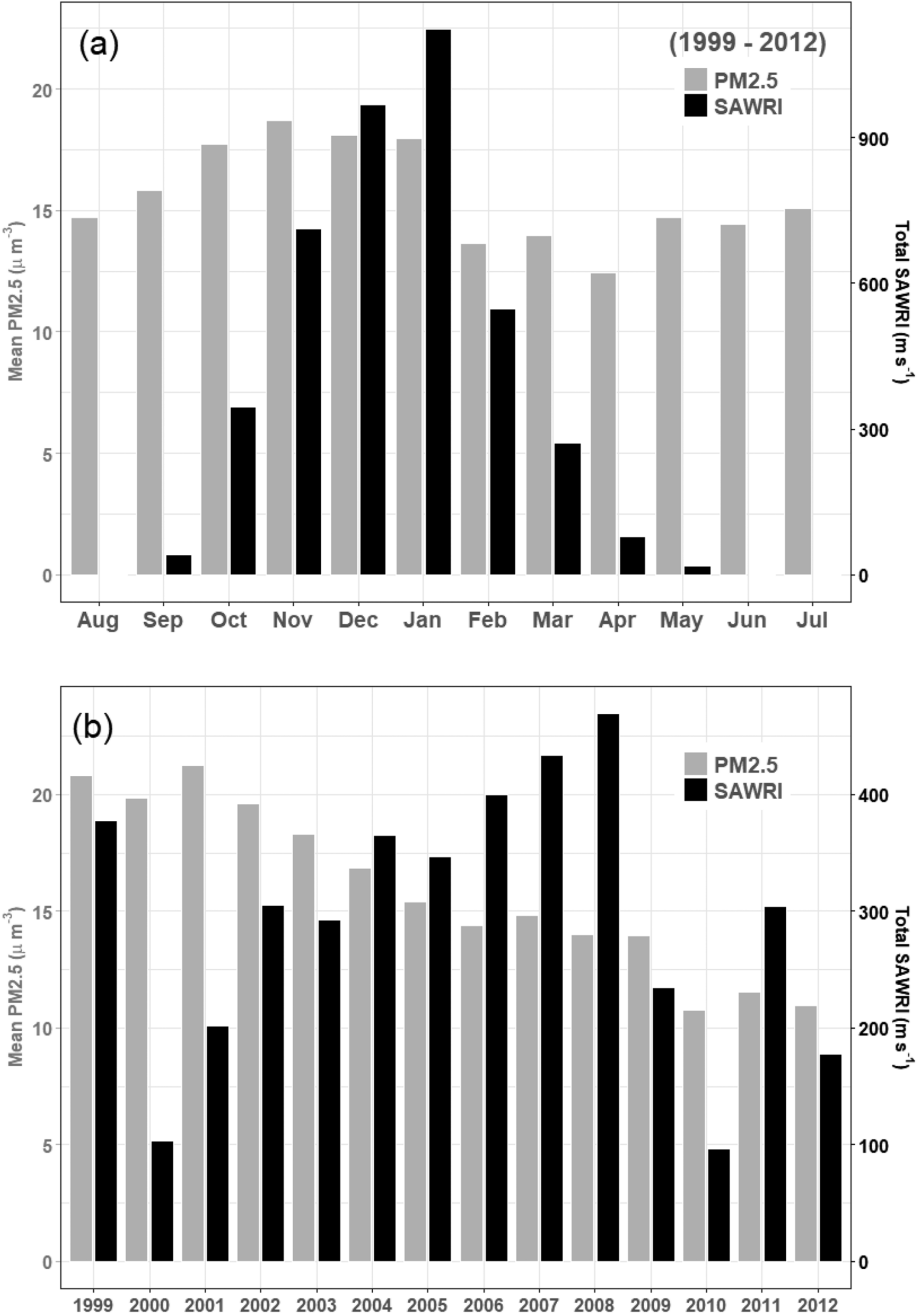
Mean PM_2.5_ and total SAWRI activity, summarized by month (a) and by calendar year (b). The annual values for PM_2.5_ consider the months of September to May of each calendar year.

For the period 1999–2012, there were 1,083 days with SAWs (i.e., SAWRI > 0). The year 2008 had the most SAW days (*n* = 125), followed by 2007 (*n* = 100). The least number of SAW days was observed in 2010 (*n* = 30) and 2,000 (*n* = 37). SAWRI reflects a liberal definition of SAW days whereby weak SAW conditions expressed over a small part of the domain make for a SAW day (SAWRI > 0), although SAWRI is very small for such days and large for strong and extensive SAWs. Total monthly regional SAW activity (i.e., the sum of SAWRI over a month, reflecting both intensity and frequency of SAW; Figure 1) peaks between the months of November and January. We summarized daily SAWRI values per month and per calendar year (Tables S1 and S2). The overall maximum regional wind speeds for the strongest SAW events on record were observed in October 2007. These regional average values, ranging from 12.7 to 13.4 m/s can be described as extreme (>10 m/s), being in the 10% of the strongest SAWRI values during a 65‐year record (Guzman‐Morales et al., 2016). The same summary but for daily mean PM_2.5_ values by month (September to May, the SAW season) showed maximum and average concentrations peaking in fall and winter months, with an exceptional maximum value for the 24‐hr levels in October 2003 (237.3 μg/m^3^), the month/year with the highest wildfire activity (i.e., acres burned) in SoCal and moderate‐intensity (albeit long‐duration) SAWs (i.e., SAWRI ranging between 5 and 10 m/s; Guzman‐Morales et al., 2016). This supports the notion that even moderately strong SAWs can cause extreme fire spread.

### 3.2 Dichotomous Effects of SAW on Daily PM_2.5_

**Figure 2:**
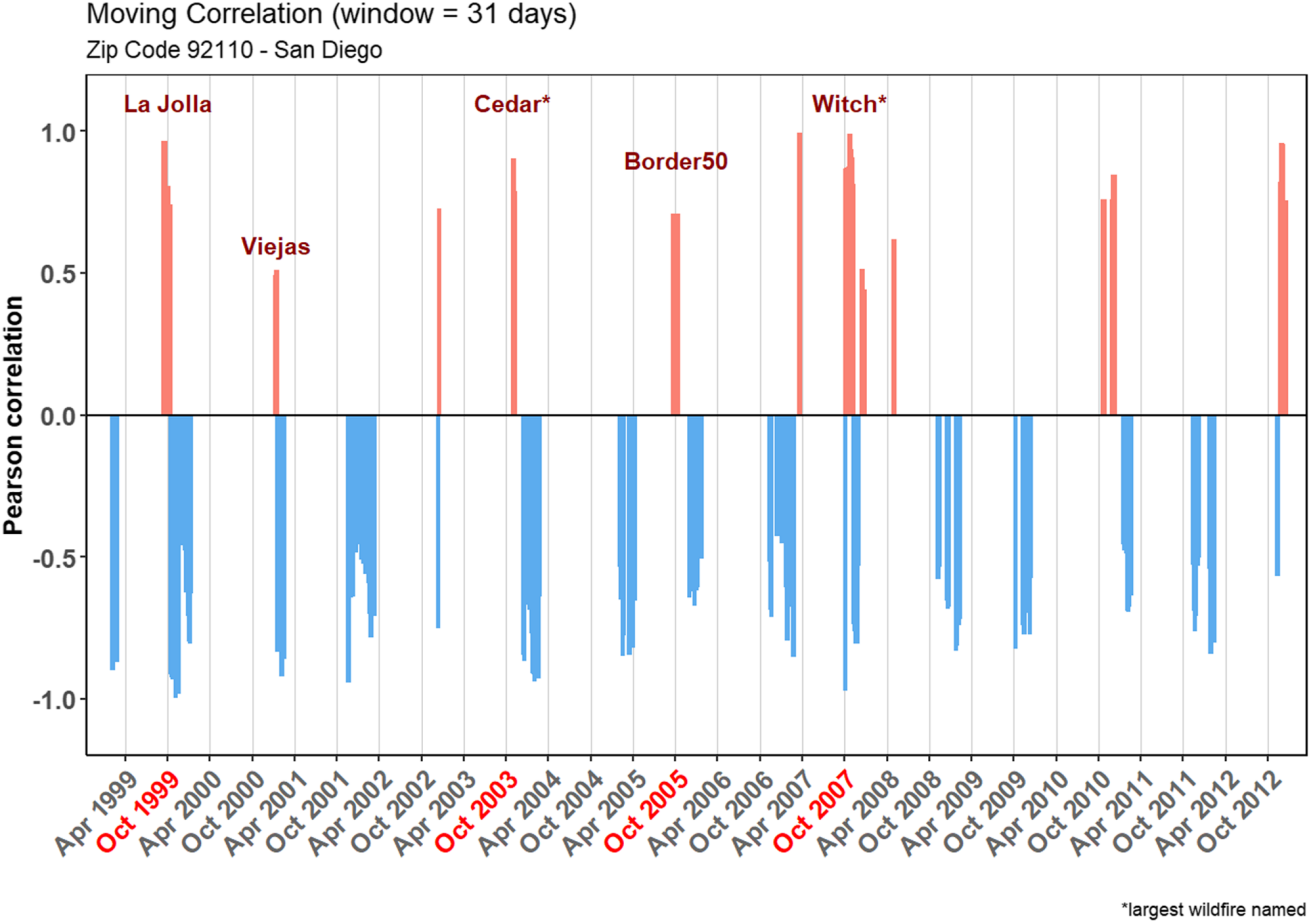
Significant positive (red) and negative (blue) correlations in a San Diego coastal zip code (location shown in Figure S2 in the supporting information). Dates for positive correlations associated with wildfires upwind are highlighted in red; in addition, the Viejas Fire burned 11,000 acres in January 2001. Other wildfire names are also included to highlight the contribution of smoke to particular positive correlations.

SAWs predominantly reduce daily PM_2.5_ as evidenced by the negative correlations during typical SAW seasons (blue bars in Figure 2, showing a coastal zip code as an example). They do so by transporting pollution particles offshore. However, intermittently, SAWs have the opposite—polluting—effect when significant positive correlations with PM_2.5_ (red bars in Figure 2) are observed. Below, we show that this occurs mostly on days with wildfires upwind, with SAWs supporting wildfire growth and advecting smoke toward coastal areas. This dichotomous effect of SAW thus allowed us to identify zip codes affected by episodic, wildfire‐related PM_2.5_.

**Figure 3:**
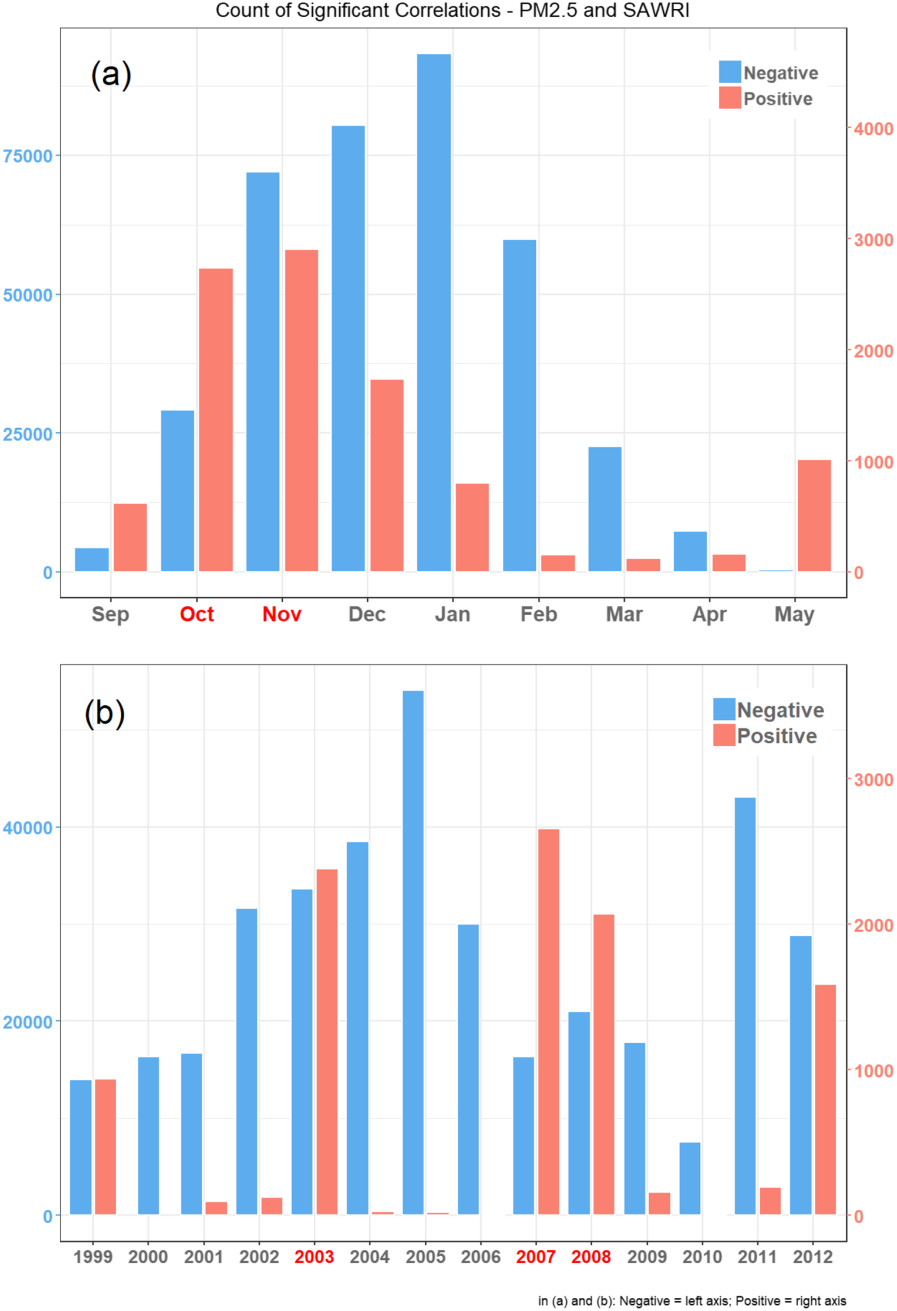
Count of significant positive and negative correlations per day/zip code, summarized per (a) month and (b) calendar year. Months and years among the largest burned areas (Tables S1 and S2) are highlighted in red. Note the different scales for the positive and negative correlation counts.

Examining the frequency of counts of significant correlations between SAWRI and PM_2.5_ for all SAW days and zip codes resolved in this study, we found that the highest number of negative correlations occurred during the months of November, December, January, and February and during years devoid of large wildfires, for example, 2004, 2005, and 2011 (Figure 3). This result reflects the ventilation effect of SAW during the winter peak of the SAW season, when SAWs are most frequent and typically not associated with wildfires after the start of the rainy season. Positive correlations were most frequent in October and November and in years 2003, 2007, and 2008 (Figure 3b), when autumn wildfires were numerous and widespread across Southern California (Tables S1 and S2).

**Figure 4:**
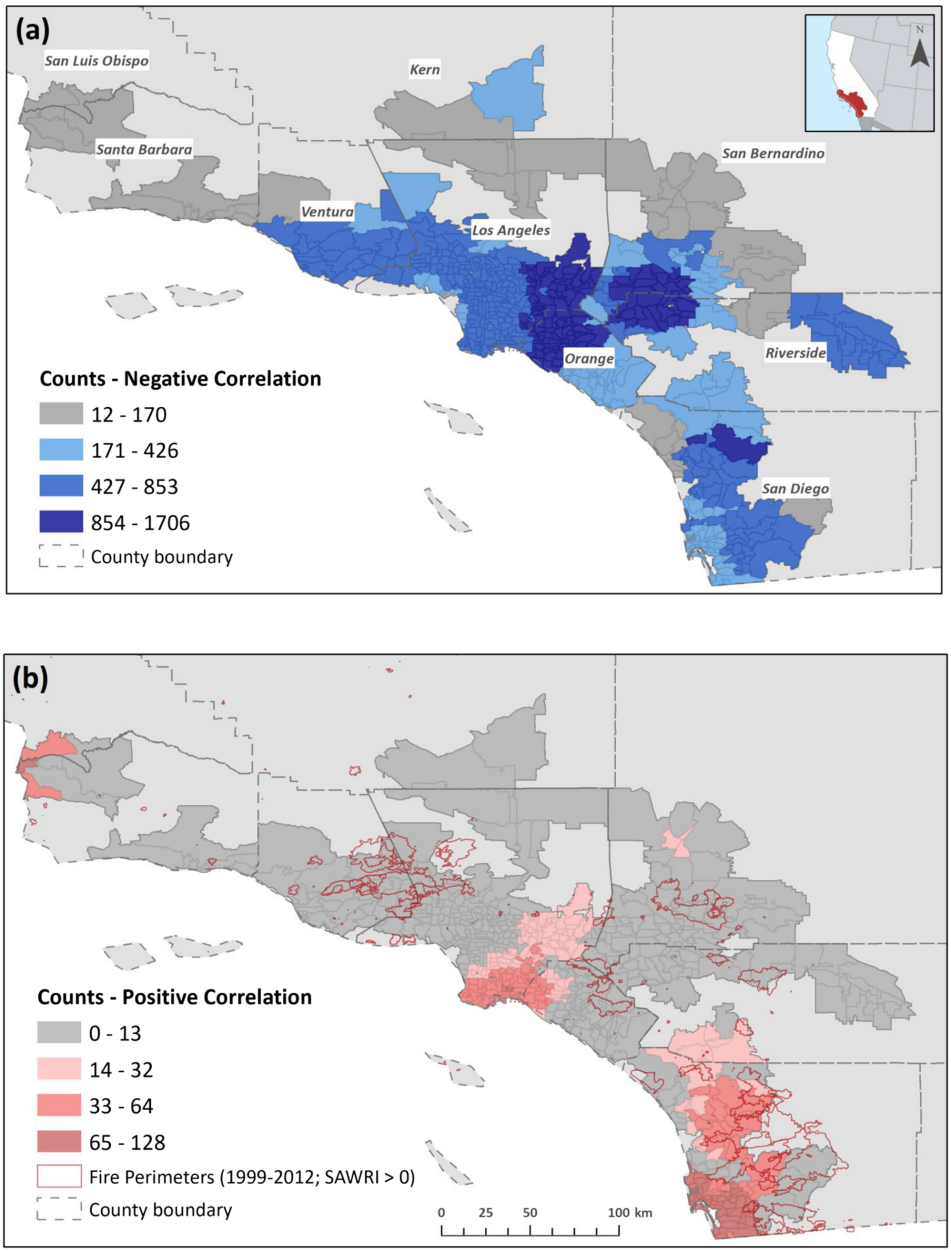
Total counts of negative (a) and positive (b) significant correlations per zip code. Fire perimeters obtained from the Fire and Resource Assessment Program of the California Department of Forestry and Fire Protection (http://frap.fire.ca.gov/) display the total area burned, and the date reflects the start of the fire.

Spatially, inland zip codes (in Los Angeles, Riverside, San Bernardino, and north San Diego counties) and those in the northern half of Orange County had the highest number of negative correlations (Figure 4a), followed by areas surrounding large cities (i.e., Los Angeles and San Diego), though these were also the areas with the largest number of air quality observations (Figure S2). On the other hand, the polluting impact of SAW and wildfires was mainly observed in coastal zip codes (Figure 4b) where SAWs spread PM_2.5_ in smoke plumes, thus causing damage to the health of a large and diverse population in terms of socioeconomic and demographic conditions (Hutchinson et al., 2018; Thelen et al., 2013).

### 3.3 Case Study: Santa Ana‐Driven Wildfire Outbreak of October 2007

**Figure 5:**
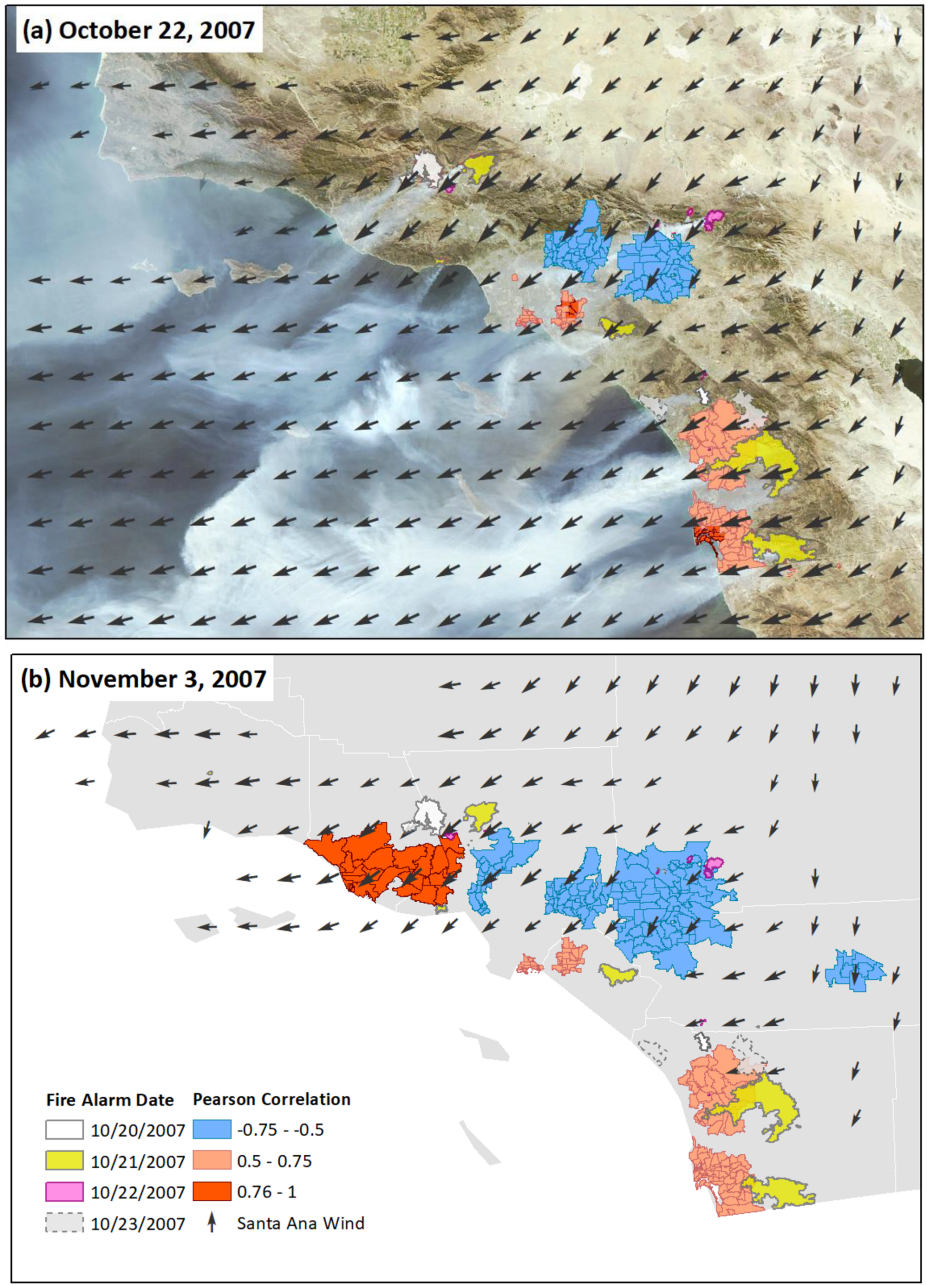
Case study highlighting significant correlation between PM_2.5_ and wildfires. Daily gridded SAW vectors shown were obtained from Guzman‐Morales et al., 2016. (a) The Moderate Resolution Imaging Spectroradiometer Rapid Response System (https://lance.modaps.eosdis.nasa.gov/cgi-bin/imagery/gallery.cgi) satellite image shows the smoke plumes for fires burning on 22 October, and wind vectors represent wind velocity for that same day. High positive correlations are found in coastal zip codes, which remained with poor air quality conditions after (b) 2 weeks from the onset of the first wildfire. Fire perimeters display the total area burned and the date reflects the start of the fire.

Figure 5 shows an example of the relationship between extreme SAW and PM_2.5_ during a series of wildfires across SoCal starting 20 October 2007. Figure 5a displays positive correlations between the two variables in coastal zip codes. Together with the satellite image in Figure 5a, these results illustrate the detrimental effect of winds on PM_2.5_ levels where smoke from wildland fires was transported toward the most populated coastal regions (Figure S3). This was the strongest SAW event on our 14‐year record, although not unprecedented in a longer SAW record (Guzman‐Morales et al., 2016). The negative correlations observed in inland zip codes account for the initial ventilation effect at the onset of SAW around 21 October and before the fires marked much of SoCal by 22 October.

**Figure 6:**
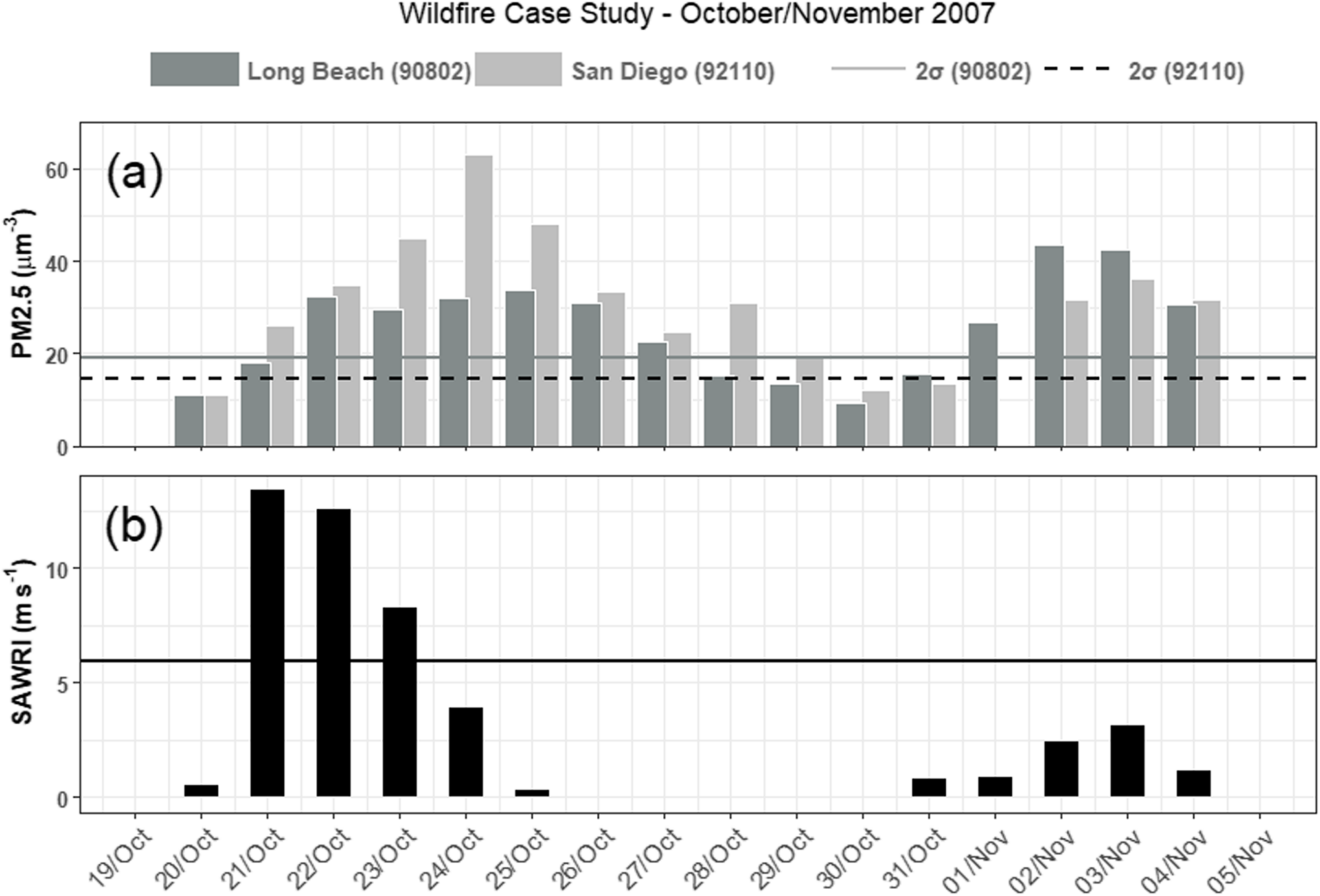
(a) PM_2.5_ concentrations for coastal zip codes in Long Beach (in Los Angeles County) and San Diego (in San Diego County). (b) SAWRI for the 2‐week period of active wildfires during October to November 2007.

Two weeks after wildfire onset (Figure 5b), more inland zip codes started to show a significant negative relationship (i.e., a ventilation effect of the second—Figure 6b—SAW event of the period), while the positive correlations remained strong and widespread in the coastal zone. Figure 6a shows levels of PM_2.5_ in two coastal zip codes peaking 4–5 days after the onset of the first wildfires upwind. This is when the strong initial SAW event was subsiding and the accumulating smoke lingered at the coast.

## 4 Discussion and Conclusions

Our study shows that the heterogeneity in the inland‐coastal spatial patterns of PM_2.5_ in SoCal is enhanced by SAW, which has been previously acknowledged though not explicitly examined (e.g., Choi et al., 2013). SAWs have a predominant negative relationship with PM_2.5_, which translates into a beneficial (ventilation) effect as background pollutants are transported offshore and away from inland and coastal areas. This ventilation effect of SAW might be more visible in inland areas since they are typically among the most polluted given pollution sources, topography, and prevailing westerly winds. Though SAWs have been anecdotally linked to improved air quality conditions in terms of visibility and reduction of haze (Corbett, 1996), our study provides evidence of the ventilation effect on PM_2.5_.

The opposite (polluting) effect is observed mainly when wildfires occur; namely, SAWs contribute to air pollution by transporting smoke PM_2.5_ from inland areas, where wildfires are spread by SAW, particularly to the densely populated coastal zone. Fine particulate matter levels exceeding the 24‐hr national standard of 35 μg/m^3^ for PM_2.5_, have been associated with specific wildfire events in the region (Phuleria et al., 2005; Wu et al., 2006; Kochi et al., 2016) with detrimental impacts on human health (e.g., Delfino et al., 2009; Hutchinson et al., 2018).

Limitations such as differing measurement frequency and time period covered in observational PM_2.5_ data might have affected our analysis in certain areas with fewer observations. For instance, most monitoring stations found in Ventura and Santa Barbara counties have recorded concentrations at 3‐day intervals, with a few of these changing to daily frequency starting 2010. This, in particular, could have precluded detecting the impact of SAWs on PM_2.5_ in the presence of large wildfires (i.e., Ventura County, Figure 4b) in 2003 and 2007. Monitors located in and in the proximity of highly urbanized areas in Los Angeles, Orange and San Diego counties tend to include daily measurements of PM_2.5_ and for the longest time periods within our study framework (1999–2012). In addition, SAWRI is a regional index which does not reflect spatial particularities, for example, in areas such as western Santa Barbara County where SAWs are typically not particularly strong.

Simple statistical approaches quantifying relationships between wind and PM_2.5_ in the presence and absence of pollution sources (e.g., wildfire), could be particularly useful to help in forecasting and identifying wildfire‐generated PM_2.5_. The same approach used here could be extended to other downslope wind systems in California and elsewhere, which also spread wildfires, for example, Diablo winds of Northern California (Mass & Ovens, 2019; Smith et al., 2018). Specific wind systems, and wind data in general, could also serve as the link between wildfire smoke PM_2.5_ and wildfire risk in climate change scenarios (Westerling, 2018).

Future work in relation to SAW and PM_2.5_ pollution events during and following wildfires will assess health risks from wind‐blown smoke associated with historical wildfires. Wildfire activity is already on the rise in California due to anthropogenic causes including global climate change (Williams et al., 2019). These regional trends are consistent with a global increase in wildfire activity (Jolly et al., 2015). Recent work suggests that wildfire severity and risk specifically in SoCal will likely intensify in the warming future, while gradually shifting from fall to winter (Williams et al., 2019). This seasonal shift is associated with a projected weakening of SAW activity, particularly in the fall and spring (that is coincident with a projected decrease in fall precipitation; Pierce et al., 2013; Swain et al., 2018; and thus more likelihood of dry fuels persisting into winter) and a sharper seasonal SAW peak in December (Guzman‐Morales & Gershunov, 2019).

The Thomas Fire that burned through most of December 2017 into January 2018, briefly becoming the largest wildfire (~ 283,000 acres) in California history, is a recent example of a late season wildfire that was made possible by a late onset of winter rains and back‐to‐back SAW events that are common at the peak of the SAW season in December. In addition, since wildfire ignitions in SoCal are mostly human‐caused, population growth and expansion of development into wildland areas increase the risk of wildfires and associated impacts (Syphard et al., 2018). California communities are attempting to mitigate these risks by, for example, considering wildfire risk in approving new housing development in the wildlands (San Diego Tribune, 2019) and implementing preventive power shutoffs by energy utilities during high fire risk conditions (New York Times, 2019). These are recent developments and more proactive measures will, no doubt, be needed.

Examination of projected health risks due to future wildfire risk and changes in SAW is warranted. Confounding the smoke‐health impacts associated with SAW and wildfires, SAW also drive coastal heat waves that incur their own health risks (Kalkstein et al., 2018), which will change in a warmer future as SAW‐driven heat waves become less frequent (Guzman‐Morales & Gershunov, 2019) but hotter (Hughes et al., 2011). The ventilation effect of SAW without wildfires, meanwhile, should be beneficial to respiratory health—a hypothesized effect to be examined and quantified in future research. Results presented here provide more impetus for a holistic study of the health impacts from SAWs on a dense and diverse coastal population, with and without wildfires, in our changing climate. Recent widespread California wildfires occurring past the data record available to us here, including the fires burning in northern and southern California at the time of this writing in the fall of 2019 and their associated air quality impacts (Los Angeles Times, 2019), provide ongoing motivation for extending this study to quantify downwind health impacts, resolving disparities, and inform more effective mitigation strategies for the future.

## Conflict of Interest

The authors declare no conflicts of interest relevant to this study.

## Supporting information



Supporting Information S1
