# Supplementary material for: Santa Ana Winds of Southern California Impact PM2.5 With and Without Smoke From Wildfires
Source: Geohealth. 2020 Jan 11;4(1):e2019GH000225. doi: 10.1029/2019GH000225 (PMC7007151; doi:10.1029/2019GH000225)
Supplement: Supplementary file 1 — Supporting Information S1 [file GH2-4-e2019GH000225-s001.docx]

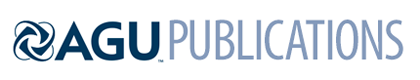


*GeoHealth*

Supporting Information for

**Santa Ana winds of Southern California impact PM2.5 with and without smoke from wildfires**

Rosana Aguilera^1^, Alexander Gershunov^1^, Sindana D. Ilango^2,3^, Janin Guzman-Morales^1^, Tarik Benmarhnia^1,2^

(1) Scripps Institution of Oceanography, University of California San Diego, La Jolla, CA, USA

(2) Department of Family Medicine and Public Health, University of California San Diego, La Jolla, CA, USA

(3) School of Public Health, San Diego State University, San Diego, CA

**Contents of this file**

Tables S1 to S2

Figures S1 to S4

**Introduction**

- Tables and additional figures that did not fit in the manuscript. Details about these results and how they were generated/used are given in the main manuscript.

| **Month** | **SAWRI [m s^-1^]** | | | **PM_2.5_ [μg m^-3^]** | | | | **Wildfires** |
| --- | --- | --- | --- | --- | --- | --- | --- | --- |
|  | *Sum* | *Max* | *n (days)* | *Min* | *Max* | *Mean (SD)* | *Median* | *Total Area (acres)* |
| Sep | 41.3 | 6.7 | 25 | 0.17 | 62.8 | 14.7 (7.6) | 13.4 | 87,524 |
| Oct | 345.0 | 13.4 | 96 | 0.16 | 237.3 | 17.2 (15.8) | 13.1 | 1,234,237 |
| Nov | 712.0 | 11.8 | 199 | 0.07 | 80.5 | 16.5 (10.3) | 13.6 | 52,697 |
| Dec | 968.0 | 12.1 | 249 | 0.21 | 84.4 | 18.5 (11.0) | 15.6 | 28,400 |
| Jan | 1123.0 | 12.2 | 263 | 0.09 | 78.1 | 18.6 (12.0) | 15.0 | 13,264 |
| Feb | 548.0 | 12.7 | 136 | 0.06 | 75.1 | 15.3 (10.0) | 12.5 | 18,862 |
| Mar | 272.0 | 11.1 | 75 | 0.07 | 41.9 | 10.8 (4.9) | 9.9 | 3,359 |
| Apr | 79.0 | 6.9 | 30 | 0.04 | 62.9 | 12.3 (5.8) | 11.8 | 3,895 |
| May | 18.2 | 3.2 | 10 | 1.90 | 42.0 | 12.1 (4.9) | 12.7 | 1,317 |

**Table S1:** Descriptive statistics for Santa Ana Wind Regional Index (SAWRI), mean PM2.5 and total area burned by wildfires, summarized per month within our 1999-2012 study period. Wildfire total burned areas related to SAWs were derived from data in Kolden and Abatzoglou (2018).

| **Year** | **SAWRI [m s^-1^]** | | | **PM_2.5_ [μg m^-3^]** | | | | **Wildfires** |
| --- | --- | --- | --- | --- | --- | --- | --- | --- |
|  | *Sum* | *Max* | *n (days)* | *Min* | *Max* | *Mean (SD)* | *Median* | *Total Area (acres)* |
| 1999 | 378 | 12.7 | 89 | 0.76 | 121.4 | 21.9 (11.9) | 18.7 | 10,916 |
| 2000 | 103 | 10.0 | 37 | 0.71 | 119.0 | 20.9 (14.6) | 16.9 | 69 |
| 2001 | 201 | 11.1 | 55 | 0.71 | 97.5 | 22.2 (13.0) | 19.2 | 18,423 |
| 2002 | 305 | 12.7 | 77 | 1.01 | 82.1 | 19.9 (11.8) | 16.9 | 46,861 |
| 2003 | 293 | 11.5 | 74 | 0.50 | 237.3 | 18.8 (13.3) | 15.3 | 665,385 |
| 2004 | 365 | 12.1 | 89 | 0.87 | 92.6 | 17.2 (10.6) | 14.4 | 3,236 |
| 2005 | 347 | 11.8 | 87 | 0.77 | 106.2 | 15.3 (9.9) | 12.8 | 40,967 |
| 2006 | 400 | 12.2 | 93 | 0.78 | 63.3 | 14.3 (8.2) | 12.4 | 73,251 |
| 2007 | 433 | 13.4 | 100 | 0.45 | 126.1 | 15.2 (10.1) | 12.3 | 491,418 |
| 2008 | 469 | 12.2 | 125 | 0.44 | 78.2 | 13.6 (7.6) | 12.6 | 63,817 |
| 2009 | 235 | 9.9 | 74 | 0.08 | 78.1 | 13.9 (7.6) | 12.4 | 26,395 |
| 2010 | 96 | 7.7 | 30 | 0.03 | 49.8 | 10.6 (5.7) | 9.5 | 1,554 |
| 2011 | 304 | 9.8 | 89 | 0.41 | 65.0 | 11.8 (6.2) | 10.6 | 244 |
| 2012 | 178 | 10.0 | 64 | 0.01 | 105.1 | 11.1 (5.9) | 10.1 | 1,017 |

**Table S2:** Descriptive statistics for Santa Ana Wind Regional Index (SAWRI), mean PM2.5 and total area burned by wildfires summarized per calendar year. PM2.5 include months with SAW activity (September-May). Wildfire total burned areas related to SAWs were derived from data in Kolden and Abatzoglou (2018).


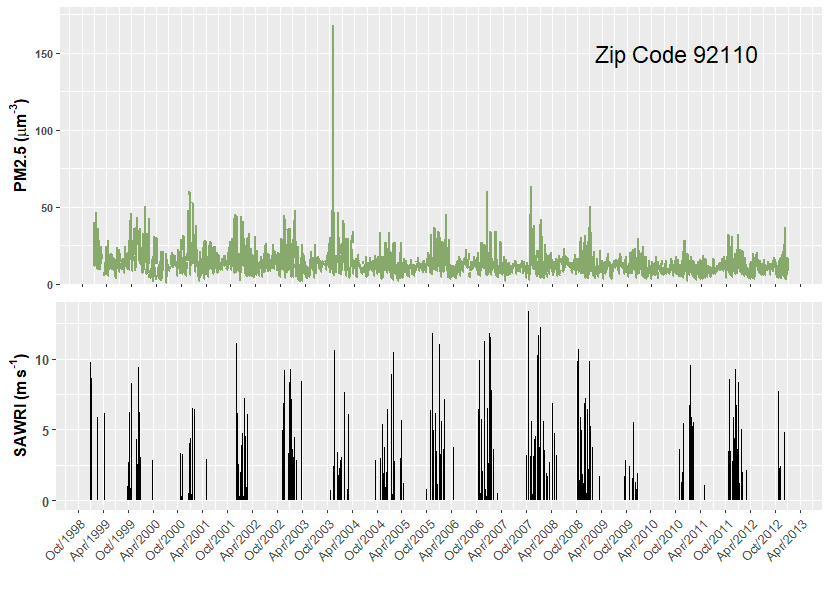


**Figure S1:** Time-series for PM2.5 levels at the San Diego 92110 Zip Code and SAWRI

**
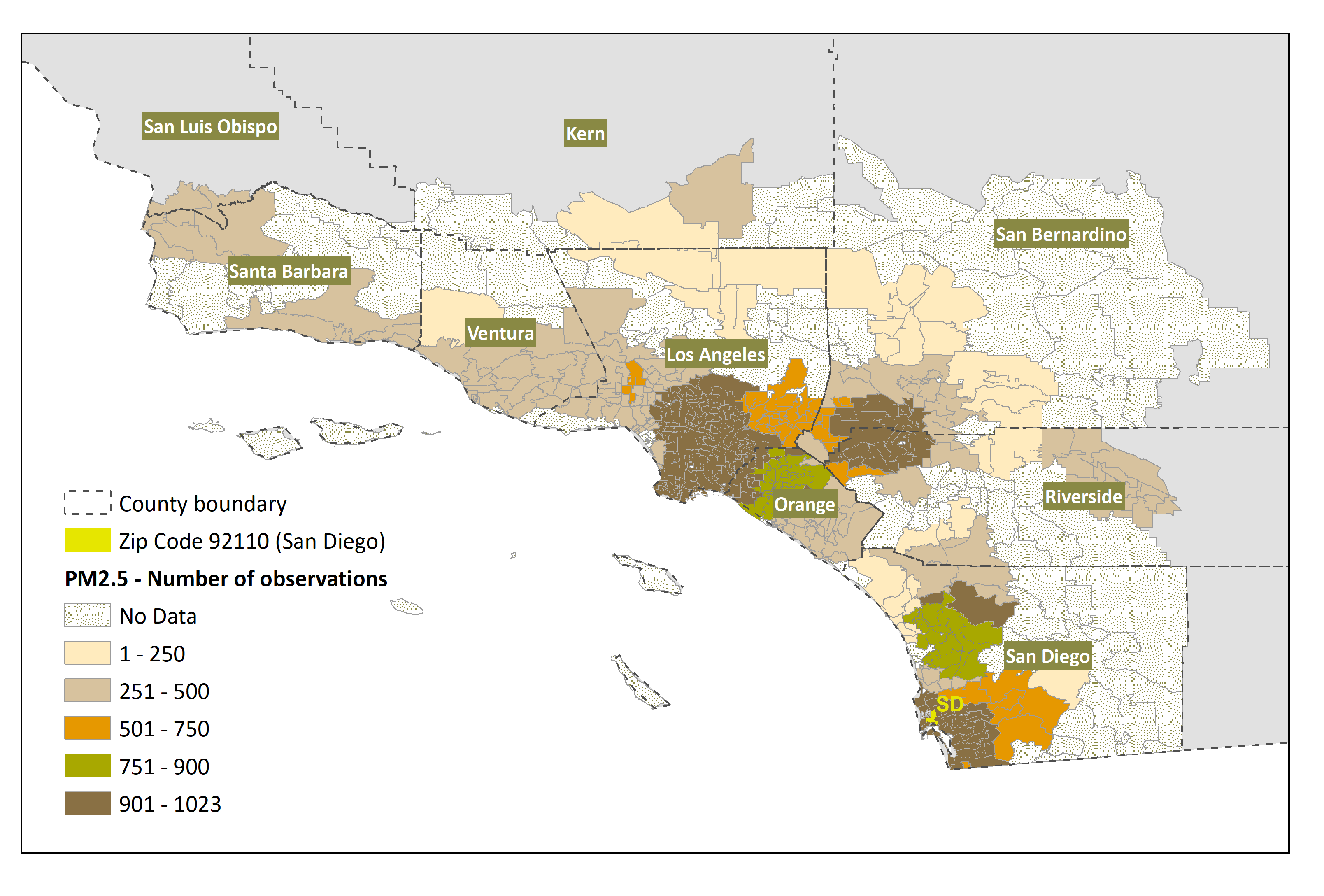
**

**Figure S2:** Counties, zip codes with data availability (number of observations for PM2.5)


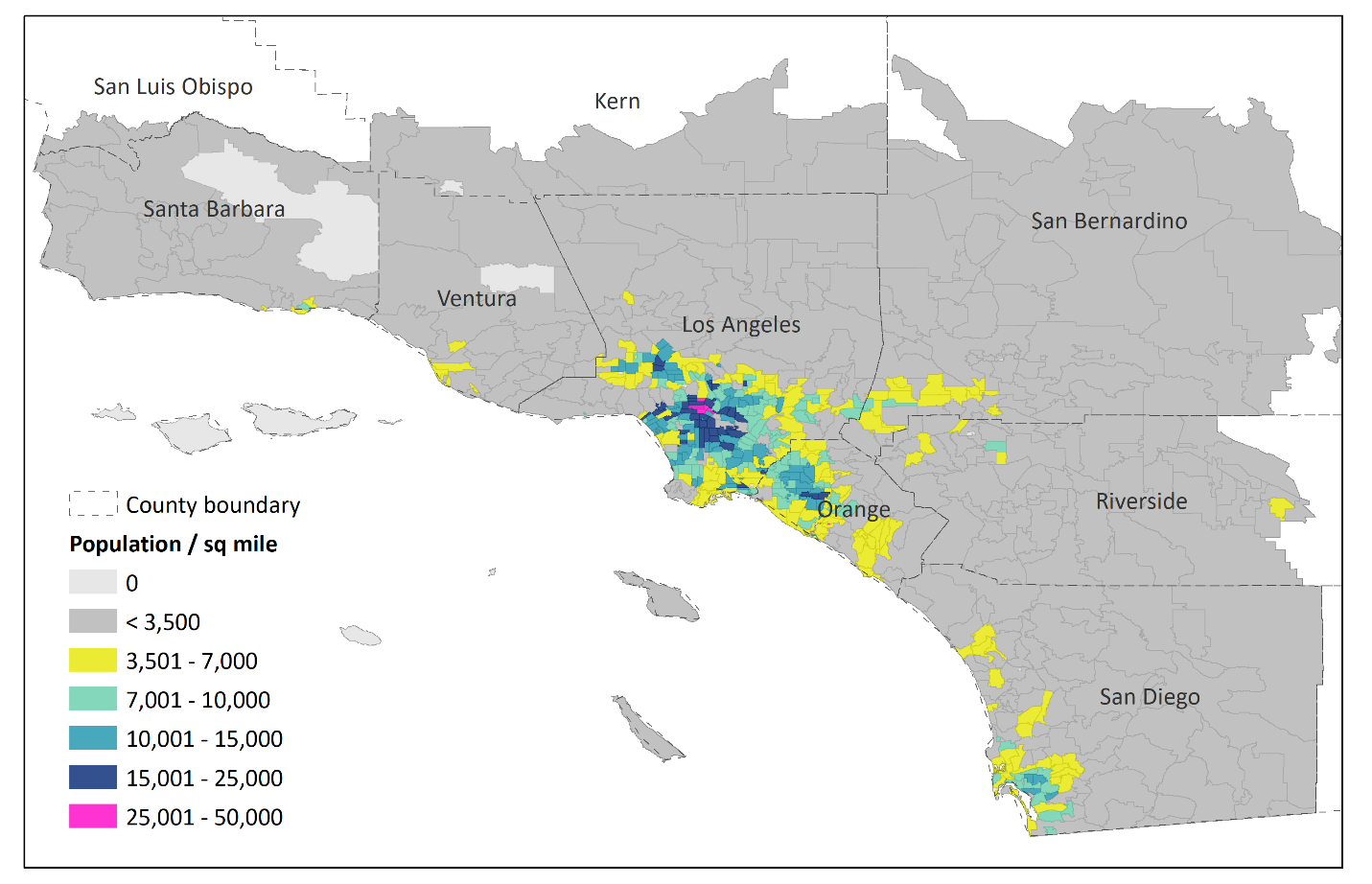


**Figure S3:** Population density (number of inhabitants per square mile)


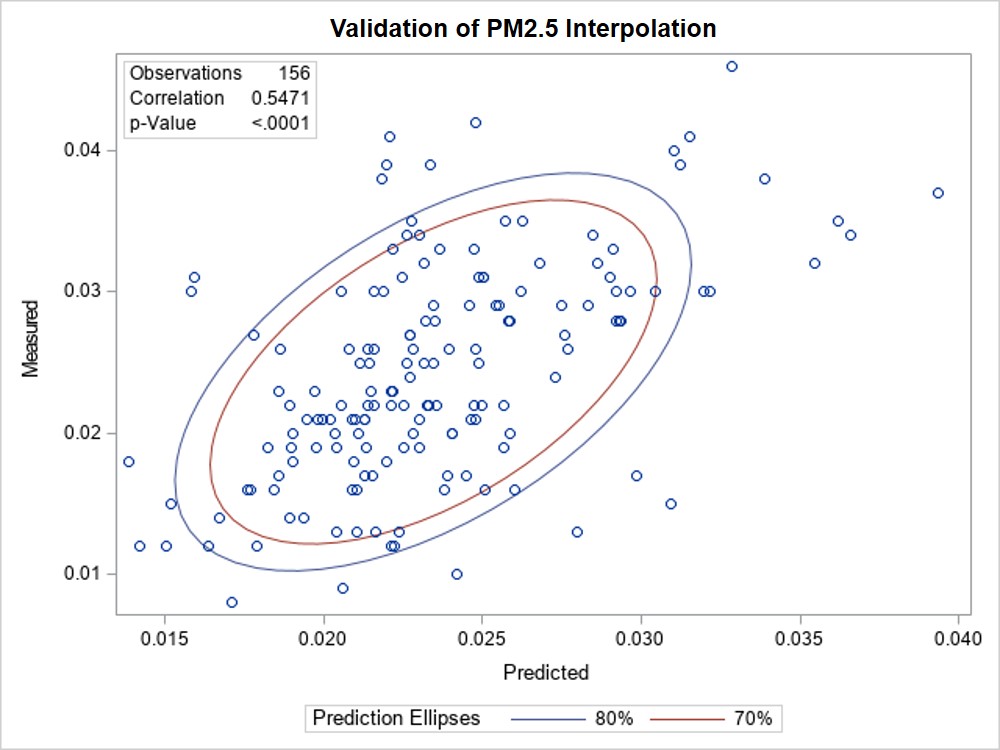


**Figure S4:** Correlation between predicted and actual values at the location of an omitted PM2.5 monitor, used to assess the validity of interpolation models at the zip code level.
